# Establishment of a multicomponent quality control method and the transfer characteristics of five markers from Qidongning Formula to rat tissues by HPLC-QQQ-MS/MS

**DOI:** 10.3389/fphar.2023.1310266

**Published:** 2023-12-05

**Authors:** Di Zhou, Jian-Ru Chen, Zi-Qi Yang, Ling Xu, Yu-Feng Huang

**Affiliations:** ^1^ Department of Oncology, Yueyang Hospital of Integrated Traditional Chinese and Western Medicine, Shanghai University of Traditional Chinese Medicine, Shanghai, China; ^2^ General Manager Office, Shanghai Tongjitang Pharmaceutical Co., Ltd., Shanghai, China; ^3^ College of Chinese Medicine, Guangzhou University of Chinese Medicine, Guangzhou, Guangdong, China; ^4^ State Key Laboratory of Traditional Chinese Medicine Syndrome, The Second Affiliated Hospital of Guangzhou University of Chinese Medicine, Guangzhou, Guangdong, China

**Keywords:** Astragali Radix, antitumour, Qidongning Formula (QDN), quality control, tissue distribution, HPLC-QQQ-MS/MS

## Abstract

**Introduction:** Traditional Chinese medicine compound preparations have become an increasingly utilized strategy for tumour treatment. Qidongning Formula (QDN) is a kind of antitumour compound preparation used in hospitals, and it can inhibit the growth of lung cancer cells. However, due to the complexity of botanical drugs, the quality evaluation of QDN is inconsistent, affecting clinical efficacy and posing potential safety risks for clinical application. Additionally, tissue distribution is an integral part of the drug development process.

**Methods:** To study the distribution characteristics of markers in compound preparations and rat tissues, a novel HPLC-QQQ-MS/MS quantitative analytical method was established to determine five markers in QDN simultaneously, and the method was verified.

**Results and discussion:** The analytical results showed that the contents of salidroside (51.6 ± 5.75 μg/g), calycosin-7-O-β-D-glucoside (94.2 ± 15.4 μg/g), specnuezhenide (371 ± 72.5 μg/g), formononetin (23.8 ± 5.39 μg/g), and polyphyllin I (87.7 ± 10.6 μg/g) were stable in different batches of QDN. After intragastric administration (13.5 g/kg) in rats for 1 h, four markers in the QDN, except polyphyllin I, were distributed in most tissues. QDN was distributed chiefly in the stomach and small intestine, followed by the liver or kidney. The study also found that specnuezhenide had the highest concentration in both QDN and rat tissues (102 ± 22.1 μg/g in the stomach), while formononetin had the highest transfer rate (0.351%) from QDN to rat intestines. The above research lays a quality research foundation for the antitumour application of QDN and provides a scientific reference for the quality control of Chinese medicine compound preparations.

## 1 Introduction

To date, the treatment of malignant tumours mainly includes radiotherapy, chemotherapy, and surgery, but the cost is high, and the side effects are significant. It has been reported that compound preparations with traditional Chinese medicine characteristics play an indispensable role in tumour treatment, and their efficacy is better than that of single-molecule drugs, which has become a new tumour treatment strategy (Wang et al., 2021). Qidongning Formula (QDN) is a kind of Chinese medicine compound prescription commonly used to treat tumours in the Oncology Department of Yueyang Hospital of Integrated Chinese and Western Medicine affiliated with Shanghai University of Traditional Chinese Medicine. QDN comprises five botanical drugs: *Astragalus memeranaceus* Bge. var. *mongholicus* Hsiao [Leguminosae: Astragali Radix], *Ophiopogon japonicus* Ker Gawl. [Liliaceae: Ophiopogonis Radix], *Ligustrum lucidum* Ait [Oleaceae: Ligustri Lucidi Fructus], *Gynostemma pentaphyllum* Makino [Cucurbitaceae: Gynostemmae pentaphylli Herba], and *Paris polyphylla* Smith var. *chinensis* Hara [Liliaceae: Paridis Rhizoma]. Clinical application and previous pharmacological studies have shown that QDN plays an antitumour role by inhibiting cell proliferation (Zheng et al., 2017) and inducing lung cancer cell apoptosis via activation of the early growth response (Zheng et al., 2017), cell cycle arrest and senescence. Our research team has also found that QDN regulates the expression of LC3B and p62 by regulating autophagy to inhibit lung cancer cell growth (Yang et al., 2019; Wang et al., 2020). However, due to the complex composition, there are deficiencies in the quality and safety control of QDN, such as a lack of research on the differences in the contents of multiple metabolites between different batches, which affects clinical efficacy and has potential safety risks for clinical application.

Traditional Chinese medicine compound preparations exert clinical efficacy through multicomponent, multitarget, and multipathway joint mechanisms. From the chemical composition, flavonoids, saponins, and iridoid glycosides are the main potential antitumour botanical metabolites of QDN. Salidroside (Wang et al., 2021), calycosin-7-O-β-D-glucoside (Dai et al., 2023), formononetin (Dai et al., 2023), and polyphyllin I (Wang et al., 2023) in QDN have been reported to have antitumour effects, as well as specnuezhenide with inhibition of oxidative stress, anti-inflammatory effects, and immunomodulatory properties (Hu et al., 2022; Tang et al., 2023). Therefore, there is an urgent need to select an appropriate method for multicomponent analysis. Liquid chromatography coupled with mass spectrometry technology is one of the most critical separation and identification analytical methods in contemporary scientific research and has the advantages of solid selectivity, high sensitivity, and good separation ability (Zhang, 2018). In this study, this technology can quantify accurately and be suitable for analysing the composition of complex systems.

In addition, tissue distribution studies are also an integral part of drug quality control (QC) and can provide important information for evaluating drug safety and efficacy. After entering the bloodstream, drugs are distributed within the blood to tissues and react with target molecules to produce biological effects (Kushida et al., 2021). Therefore, the distribution of drugs in the body is an essential factor affecting the efficacy of drugs. While the multicomponent characteristics of QDN lead to the transfer of markers into body tissues, this is a complicated study. Therefore, research on the content determination and tissue distribution of QDN helps provide a basis for improving the quality and safety control of its clinical use.

This study established a fast method to simultaneously determine the contents of five representative markers in QDN by HPLC-QQQ-MS/MS and the tissue distribution characteristics in rats. These studies provided scientific data for ensuring the stable quality of QDN and understanding the tissue distribution of each metabolite, which provided a scientific reference for the QC of Chinese medicine compound preparations.

## 2 Materials and methods

### 2.1 Chemicals and reagents

**FIGURE 1:**
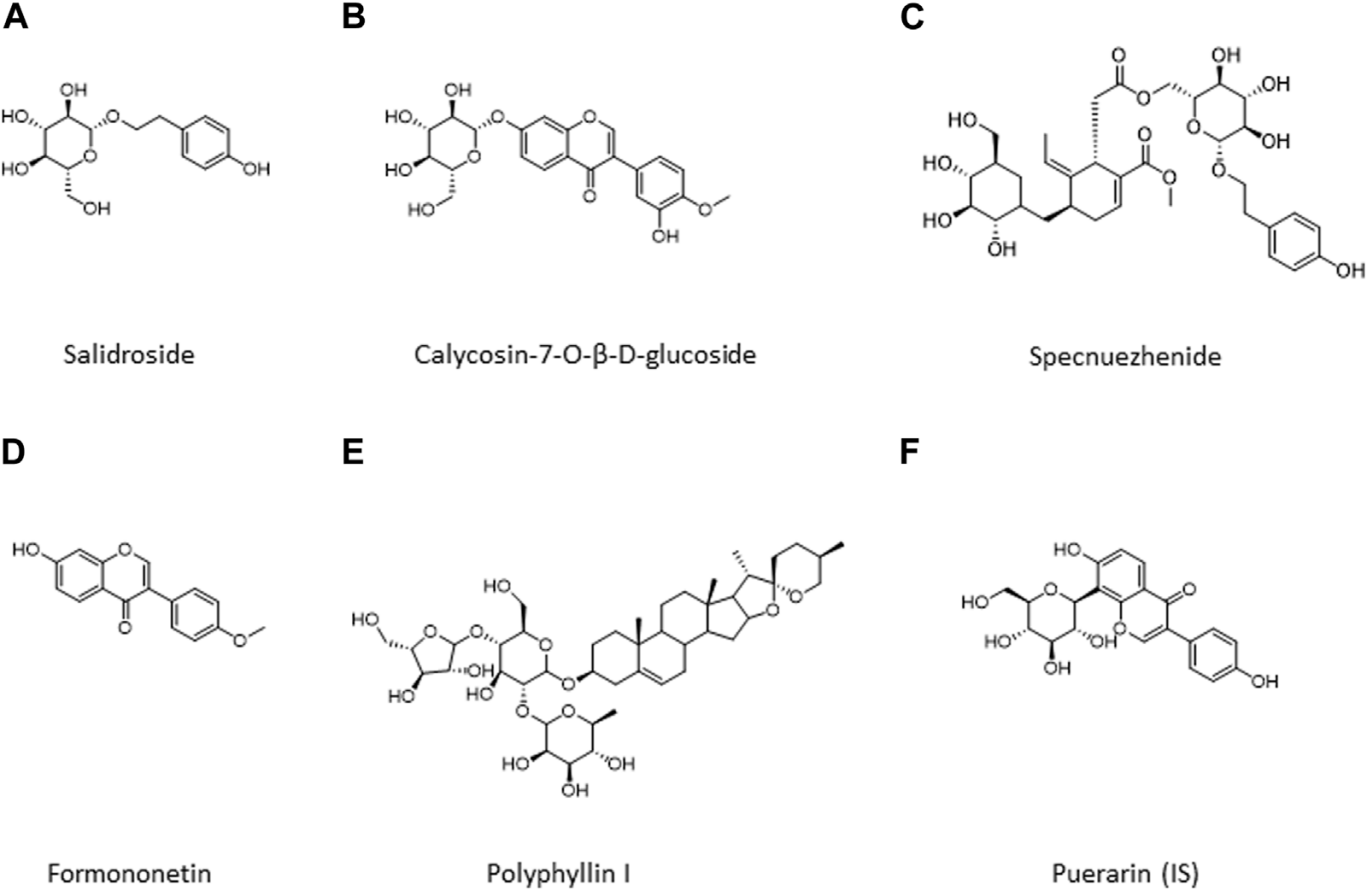
Chemical structures of five metabolites in QDN and IS. **(A)** Salidroside, **(B)** Calycosin-7-O-β-D-glucoside; **(C)** Specnuezhenide, **(D)** Formononetin; **(E)** Polyphyllin I; **(F)** Puerarin (IS).

Reference compounds of HPLC grade, i.e., salidroside, calycosin-7-O-β-D-glucoside, specnuezhenide, formononetin, and polyphyllin I, were purchased from Shanghai Yuanye Bio-Technology Co., Ltd. with purity ≥98%. Puerarin as an internal standard substance (IS) was obtained from the National Institutes for Food and Drug Control (Beijing, China). Figure 1 lists the chemical structures of these metabolites. Distilled and deionized water was prepared with a water purification system (RODI-220A1, RSJ, China).

HPLC-grade acetonitrile and methanol were obtained from AQA (Cleveland, United States) and Guangzhou Chemical Reagent Factory (Guangzhou, China). The other chemicals and reagents were of analytical grade and were commercially available.

### 2.2 QDN preparation

Botanical drugs of QDN were purchased from Yueyang Hospital of Integrated Traditional Medicine and Western Medicine affiliated with Shanghai University of Traditional Chinese Medicine. Each batch of QDN included five botanical drugs (Astragali Radix, 30 g; Ophiopogonis Radix, 10 g; Ligustri Lucidi Fructus, 10 g; Gynostemmae pentaphylli Herba, 15 g; and Paridis Rhizoma, 10 g). Each botanical drug was identified by Chen Jianru, a chief pharmacist of Chinese medicine from Shanghai Tongjitang Pharmaceutical Co., LTD., and conformed to the relevant provisions of the Pharmacopoeia of the People’s Republic of China (Part 1, 2020 edition). The extraction and processing procedure were as follows: a certain amount of botanical drugs was weighed according to the prescription ratio, decocted twice with 6–8 times water for 40 min each time, and filtered. The combined filtrate was rotary evaporated at 80°C and concentrated to a raw drug mass concentration of 1.5 g/mL and stored at 4°C for the next step of the experiment.

A total of five batches of QDN samples were prepared. The concentrated liquid of the samples was taken from 200 μL, added to 800 μL methanol, vortexed, and centrifuged at 12,000 rpm for 10 min; the supernatant was obtained and then passed through a 0.22 μm filter membrane for LC‒MS content determination.

### 2.3 Chromatographic conditions

For content determination and tissue distribution study, the HPLC-QQQ-MS/MS system was performed on an Agilent 1,260 series HPLC system combined with an Agilent 6,460 Triple Quadrupole Mass (Agilent Technologies, United States) equipped with an electrospray ionization (ESI) source.

Chromatographic separation was carried out on a Waters Acquity UPLC T3 column (2.1 mm × 100 mm, 1.7 μm) (Waters, United States), and the column temperature was maintained at 30°C. The mobile phase was composed of water containing 0.1% formic acid (A) and acetonitrile (B) with the following gradient elution program: 0–2.5 min, 20% B; 2.5–5 min, 50% B; and 5–10 min, 90% B. The flow rate was set at 0.25 mL/min. The autosampler was set at 4°C, and the injection volume was 5 μL.

### 2.4 Mass spectrometric conditions

The mass spectrometer was acquired in positive mode with a MassHunter workstation software optimizer (Agilent Technologies, United States) for the detection of salidroside, calycosin-7-O-β-D-glucoside, specnuezhenide, and polyphyllin I. Formononetin and IS data were obtained in negative ESI mode. Quantification was performed in multiple reaction monitoring (MRM) mode. The MRM transition was *m/z* 318.15 → 85.00 for salidroside, *m/z* 447.13 → 285.00 for calycosin-7-O-β-D-glucoside, *m/z* 704.27 → 165.00 for specnuezhenide, *m/z* 267.06 → 252.00 for formononetin, *m/z* 855.48 → 85.00 for polyphyllin I, and *m/z* 415.10 → 267.00 for IS. Other optimized operation parameters were as follows: nebulizer, 15 psig; ion spray voltage, 4,000 V; capillary temperature, 300 °C; dry gas (N_2_) flow rate, 11.0 L/min; fragmentor voltage, 85 V (salidroside), 120 V (calycosin-7-O-β-D-glucoside, specnuezhenide, and formononetin), and 155 V (IS); collision energy, 22 V (salidroside), 14 V (calycosin-7-O-β-D-glucoside and formononetin), 38 V (specnuezhenide), and 34 V (IS).

### 2.5 Standard and sample preparation

#### 2.5.1 Preparation of stock solutions, calibration standards, and quality controls

The stock solutions of salidroside (5.25 mg/mL), calycosin-7-O-β-D-glucoside (1.74 mg/mL), specnuezhenide (5.80 mg/mL), formononetin (1.63 mg/mL), and polyphyllin I (6.71 mg/mL) were prepared with methanol.

A series of calibration standards were diluted with methanol in equal proportions and set at seven levels, with the highest concentration at 52.5 μg/mL for salidroside, 87.0 μg/mL for calycosin-7-O-β-D-glucoside, 290 μg/mL for specnuezhenide, 3.26 μg/mL for formononetin, and 67.1 μg/mL for polyphyllin I. All stock and working solutions were kept at 4°C.

QC samples at three concentration levels (low: 5% of the highest calibration concentration; medium: 15% of the highest calibration concentration; high: 80% of the highest calibration concentration) were prepared similarly for the tissue distribution study. All of the calibration standards and QC samples were freshly prepared before use.

#### 2.5.2 Tissue sample collection and preparation

In the present study, the experimentation on rats was approved by an independent ethics committee at Guangdong Provincial Hospital of Chinese Medicine. The experiment was performed in an SPF laboratory authorized by the Guangdong Provincial Government. Six SPF SD rats (220 ± 20 g, three males and three females), fasted 12 h before administration but given free access to drinking water, were given intragastric administration of QDN (13.5 g/kg, equivalent to twice the clinical dose of QDN) at a dose of 10 mL/kg. One hour after administration, the rats’ organs, namely, the heart, liver, spleen, lung, kidney, stomach, small intestine, and brain, were removed under anaesthesia and rinsed with PBS. The wet weight of each tissue was accurately weighed at approximately 0.2 g, and 25 μL puerarin solution (2.04 μg/mL) was added as an IS.

For each tissue sample, 200 μL PBS and 800 μL acetonitrile were added to the centrifuge tube, homogenized, and centrifuged at 12,000 rpm for 10 min. The supernatant was taken and dried under nitrogen. Then, 100 μL methanol was added to the residue, which was vortexed for 3 min, dissolved by ultrasound, and centrifuged at 13,500 rpm for 10 min, and 5 μL supernatant was collected for LC‒MS analysis. Another drug-free rat was selected and treated as described above as the corresponding blank group.

### 2.6 Method validation

The methodologies of content determination were validated by the guidelines for Validation of Quality Standards of Traditional Chinese Medicine (China Pharmacopoeia Commission, 2020), while the methodological investigation of tissue samples was conducted according to the Guidance for Nonclinical Pharmacokinetics of Medicinal Products (Center for Drug Evaluation of National Medical Products Administration, 2014). Method validation, including specificity, linearity, precision, accuracy, repeatability, stability, recovery and matrix effect, was well studied in the Supplementary Material for details.

### 2.7 Data analysis

Mass data analysis was carried out by MassHunter Workstation software Quantitative Analysis (Agilent Technologies, Santa Clara, United States). The concentrations were evaluated via linear regression analysis. The transfer rates were calculated by the following equations: transfer rate (%) = (metabolite content in tissue × tissue weight)/(metabolite content in QDN × QDN weight) ×100%. All data are expressed as the mean ± standard deviation (SD).

## 3 Results

### 3.1 Method validation

#### 3.1.1 Specificity

**FIGURE 2:**
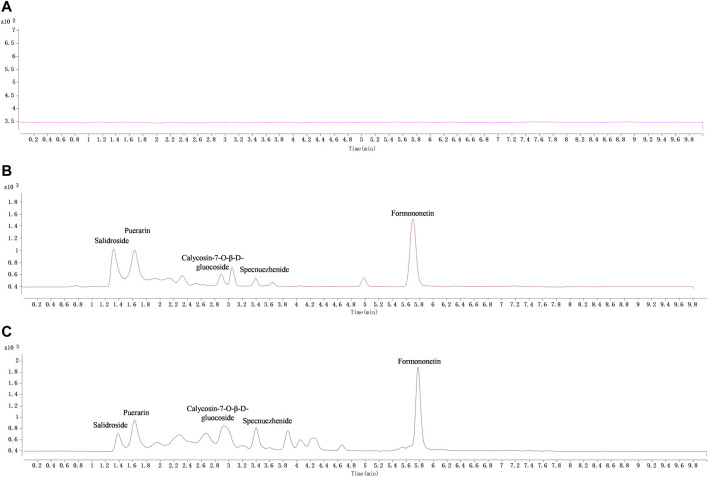
The typical MRM chromatograms of QDN and IS in liver samples. **(A)** Blank liver sample, **(B)** blank liver spiked with the mixed reference solution and IS (puerarin, 2.04 μg/mL), **(C)** liver sample collected at 1 h after i. g. administration of QDN and spiked with IS.

Under the newly established chromatographic conditions, the peak shape of each target metabolite to be measured in tissues was well separated, and there was no interference between the matrix and IS. The MRM diagram is shown in Figure 2.

#### 3.1.2 Linearity

The linear equations, correlation coefficients (r), linear ranges, and quantitative limits of each metabolite are shown in Tables 1, 2. The results exhibited good linearity of metabolites at their respective linear ranges, which met the criteria of drug analysis.

**TABLE 1:** The linearity, LODs, and LOQs of five metabolites in the QDN.

Analyte	Linearity			LOD	LOQ
	Calibration curve	r	Range (μg/mL)	(μg/mL)	(μg/mL)
Salidroside	*Y* = 549.7665*X*+778.055	0.9997	3.28–52.5	0.109	0.328
Calycosin-7-O-β-D-glucoside	*Y* = 12762.7512*X*+38867.0532	0.9999	5.44–87.0	0.0136	0.0408
Specnuezhenide	*Y* = 822.4322*X*+8950.6137	0.9999	18.1–290	0.252	0.755
Formononetin	*Y* = 921.4881*X*+88.2797	0.9999	0.204–3.26	0.0313	0.0625
Polyphyllin I	*Y* = 164.5261*X*+136.4306	0.9999	4.19–67.1	0.700	2.10

**TABLE 2:** The linearity, LODs, and LOQs of the four metabolites in the QDN in liver homogenate.

Analyte	Linearity			LOD	LOQ
	Calibration curve	r	Range (μg/mL)	(μg/mL)	(μg/mL)
Salidroside	*Y* = 69.17*X*+0.2876	0.9960	4.10–263	0.123	0.410
Calycosin-7-O-β-D-glucoside	*Y* = 4.44*X*+0.00487	0.9960	0.0544–3.48	0.0163	0.0544
Specnuezhenide	*Y* = 0.36*X*+0.1076	0.9975	0.906–58.0	0.272	0.906
Formononetin	*Y* = 19.71*X*+0.40337	0.9950	0.0637–4.08	0.0191	0.0637

#### 3.1.3 Precision and accuracy

The precision results of the five metabolites in the QDN are summarized in Table 3. The results of the inter- and intraday precision (RSD%) and accuracy (RE%) of the four metabolites in the biological matrix are listed in Table 4. Both RSDs and REs results in these tables indicated that the method was feasible to analyse QDN compound prescription and biological samples.

**TABLE 3:** Precision, repeatability, stability, and recovery of five metabolites in QDN (*n* = 6).

Analytes	Precision (RSD, %)	Repeatability (RSD, %)	Stability (RSD, %)	Recovery	
				Mean ± SD (%)	RSD (%)
Salidroside	2.69	3.34	3.73	99.2 ± 3.29	3.32
Calycosin-7-O-β-D-glucoside	1.10	0.661	2.82	99.0 ± 2.63	2.65
Specnuezhenide	2.95	4.72	4.94	98.9 ± 2.57	2.60
Formononetin	2.02	1.48	3.15	101 ± 2.04	2.02
Polyphyllin I	1.87	1.76	4.20	99.7 ± 3.42	3.43

**TABLE 4:** Precision, accuracy, extraction recovery, and matrix effect of four metabolites in the QDN in liver homogenate (*n* = 6).

Analyte	Theoretical conc	Intraday			Interday			Recovery		Matrix effect	
	(mg/mL)	Conc. (μg/mL)	RSD (%)	RE (%)	Conc. (μg/mL)	RSD (%)	RE (%)	Mean ± SD (%)	RSD (%)	Mean ± SD (%)	RSD (%)
Salidroside	0.00525	0.00544 ± 0.000396	7.27	3.62	0.00527 ± 0.000222	4.21	5.02	102 ± 7.91	7.75	106 ± 4.82	4.54
	0.0394	0.0389 ± 0.00395	10.16	−1.22	0.0407 ± 0.00238	5.84	−6.19	101 ± 5.63	5.56	89.6 ± 3.34	3.73
	0.210	0.198 ± 0.00687	3.47	−5.76	0.212 ± 0.0136	6.43	−0.37	107 ± 8.02	7.48	98.4 ± 2.04	2.07
Calycosin-7-O-β-D-glucoside	0.0696	0.0641 ± 0.00202	3.15	−7.90	0.0672 ± 0.00363	5.40	−5.61	95.3 ± 2.25	2.36	97.0 ± 2.22	2.29
	0.522	0.529 ± 0.0251	4.75	1.30	0.518 ± 0.0191	3.69	4.44	101 ± 3.95	3.90	98.0 ± 0.560	0.571
	2.78	2.89 ± 0.309	10.71	3.74	2.94 ± 0.0472	1.61	−0.05	107 ± 11.7	11.0	102 ± 5.54	5.44
Specnuezhenide	1.16	1.21 ± 0.157	13.00	3.94	1.17 ± 0.0680	5.83	7.22	104 ± 9.64	9.29	107 ± 6.21	5.82
	8.70	8.09 ± 0.621	7.68	−7.03	8.39 ± 0.276	3.29	−0.77	99.2 ± 7.67	7.73	96.9 ± 0.735	0.759
	46.4	44.5 ± 5.34	12.02	−4.14	46.3 ± 1.65	3.56	−1.30	103 ± 11.6	11.3	97.1 ± 0.0555	0.0572
Formononetin	0.163	0.162 ± 0.00719	4.45	−0.79	0.160 ± 0.00187	1.17	−1.05	96.9 ± 2.35	2.43	99.7 ± 7.14	7.16
	1.22	1.30 ± 0.0281	2.16	6.48	1.27 ± 0.0792	6.22	−1.95	97.1 ± 5.37	5.53	93.9 ± 13.7	14.6
	6.52	6.62 ± 0.437	6.61	1.50	6.32 ± 0.261	4.13	−4.49	94.0 ± 10.3	11.0	98.8 ± 0.674	0.682

#### 3.1.4 Repeatability

The repeatability results (RSD%) of all metabolites in Table 3 were not more than 5%, indicating that the instrument had good precision for analysis and that the method was repeatable.

#### 3.1.5 Stability

The stability of the QDN in Table 3 shows that the RSD results of five metabolites with high, medium, and low concentrations in the QDN were less than 5%. The stability in liver homogenate (Table 5) showed that the RSDs of the four metabolites were less than 15% for the tissue distribution study. Both results indicated that QDN samples and biological samples were stable for quantitative determination under various experimental conditions.

**TABLE 5:** Stability of four metabolites from QDN in liver homogenate (*n* = 6).

Analyte	Theoretical conc	Room temperature for 6 h		−20°C for 30 days		Freeze‒thaw for triplicate	
	(μg/mL)	Conc. (μg/mL)	RSD (%)	Conc. (μg/mL)	RSD (%)	Conc. (μg/mL)	RSD (%)
Salidroside	0.00525	0.00489 ± 0.000403	8.24	0.00480 ± 0.000250	5.20	0.00530 ± 0.000190	3.67
	0.0394	0.0406 ± 0.00400	9.84	0.0432 ± 0.000660	1.53	0.0425 ± 0.000580	1.37
	0.210	0.205 ± 0.0291	14.2	0.227 ± 0.0192	8.47	0.211 ± 0.0110	5.19
Calycosin-7-O-β-D-glucoside	0.0696	0.0625 ± 0.00178	2.84	0.0659 ± 0.00219	3.32	0.0688 ± 0.00585	8.50
	0.522	0.516 ± 0.0273	5.28	0.525 ± 0.0256	4.87	0.528 ± 0.0234	4.43
	2.78	2.93 ± 0.294	10.0	2.90 ± 0.233	8.03	2.95 ± 0.267	9.04
Specnuezhenide	1.16	1.21 ± 0.132	11.0	1.075 ± 0.0272	2.53	1.10 ± 0.138	12.6
	8.70	8.18 ± 0.298	3.64	8.65 ± 0.778	9.00	8.56 ± 0.731	8.54
	46.4	44.8 ± 4.23	9.43	48.5 ± 5.17	10.7	47.0 ± 4.91	10.5
Formononetin	0.163	0.163 ± 0.00479	2.95	0.152 ± 0.0111	7.29	0.173 ± 0.117	6.78
	1.22	1.33 ± 0.0671	5.06	1.24 ± 0.105	8.52	1.26 ± 0.139	11.1
	6.52	6.81 ± 0.551	8.10	6.14 ± 0.641	0.641	6.10 ± 0.542	8.89

#### 3.1.6 Recovery and matrix effects

The extraction recoveries of five metabolites in QDN were in the range of 99.0%–101%, with RSD not more than 5% at three concentrations (Table 3). The average recoveries of the four metabolites in the liver were between 94.0% and 107%, with RSDs lower than 15%. The matrix effects ranged from 89.6% to 107%, with RSDs lower than 15% (Table 4). The above results suggested that the sample treatment method was consistent and reproducible.

### 3.2 Quantitative study in the QDN

**FIGURE 3:**
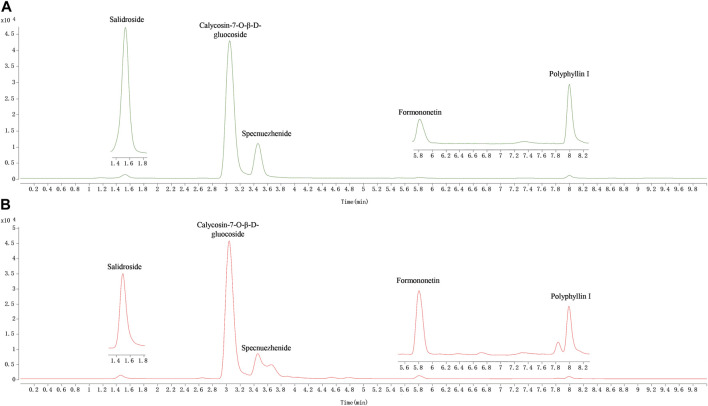
Representative MRM chromatograms of QDN. **(A)** Mixed reference solution, **(B)** QDN sample solution.

In this study, a rapid HPLC-QQQ-MS/MS method was established for the quantitative analysis of several markers in QDN as well as in rat tissue samples. The LC‒MS chromatograms of the QDN are shown in Figure 3. Five batches of QDN samples were well tested for the contents of five metabolites according to the newly established HPLC-QQQ-MS/MS method (Table 6). The average contents of salidroside, calycosin-7-O-beta-glucoside, specnuezhenide, formononetin, and polyphyllin I in the QDN were 51.6 μg/g, 94.2 μg/g, 371 μg/g, 23.8 μg/g, and 87.7 μg/g, respectively.

**TABLE 6:** The contents of five metabolites in QDN samples.

Sample	Salidroside (μg/g)	Calycosin-7-O-beta-glucoside (μg/g)	Specnuezhenide (μg/g)	Formononetin (μg/g)	Polyphyllin I (μg/g)
QDN-1	45.7	70.4	288	15.9	72.2
QDN-2	52.0	89.2	279	24.2	80.4
QDN-3	46.5	91.0	403	21.3	87.2
QDN-4	61.8	116	450	32.5	99.4
QDN-5	51.8	105	432	25.0	99.1
Mean ± SD	51.6 ± 5.75	94.2 ± 15.4	371 ± 72.5	23.8 ± 5.39	87.7 ± 10.6

### 3.3 Tissue distribution

**FIGURE 4:**
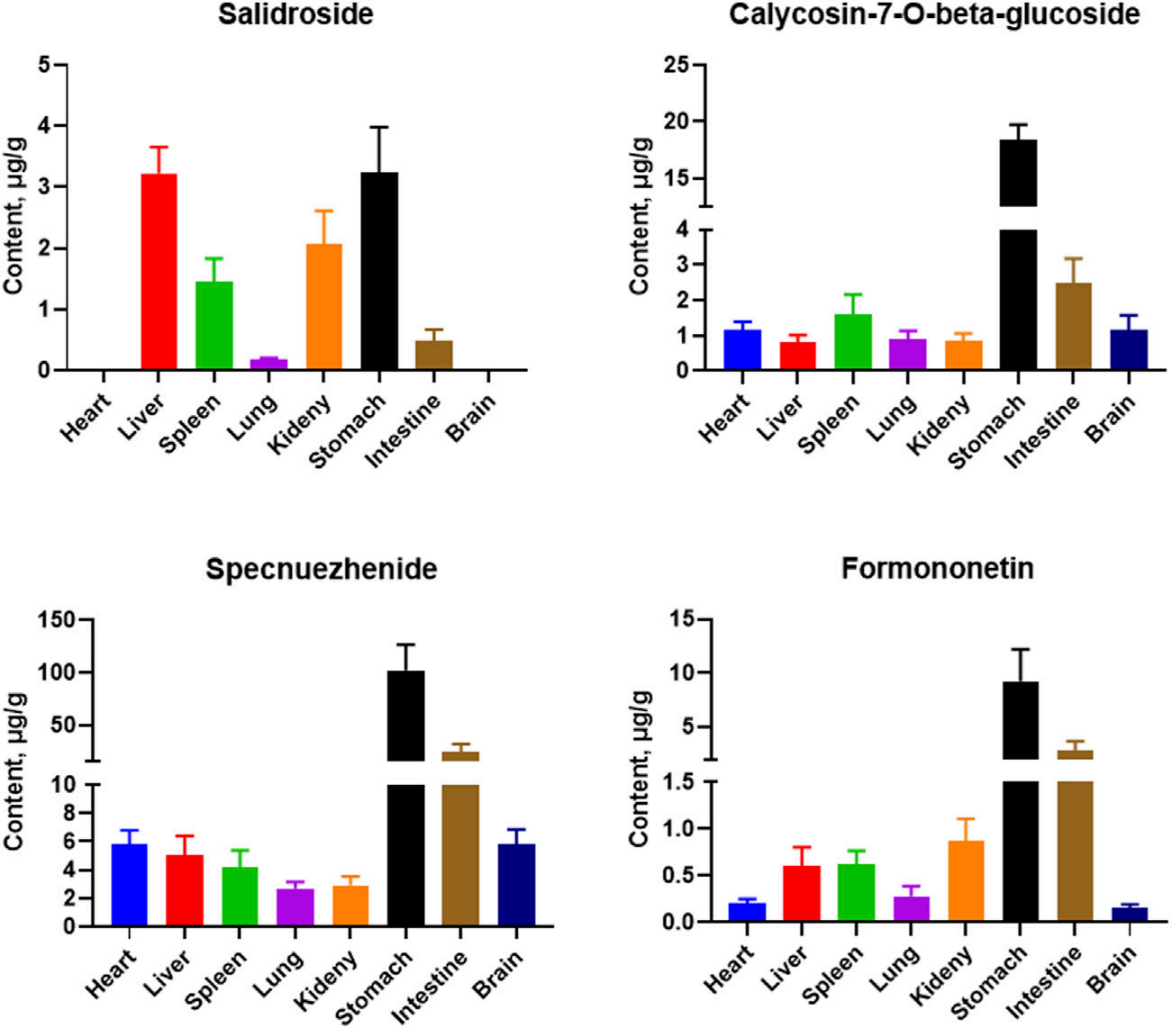
Mean concentration of four metabolites in rat tissues at 1 h after i. g. administration of 13.5 g/kg QDN. (mean ± SD, *n* = 6)

Tissue samples from the heart, liver, spleen, lung, kidney, stomach, small intestine, and brain were collected from SD rats, and the tissue distribution of five active analytes in the QDN was measured after 1 h of oral administration. Figure 2 shows the typical MRM chromatograms of QDN and IS in rat liver samples. Figure 4 shows the distribution of various metabolites in different rat tissues. Unfortunately, polyphyllin I was not found in rat tissues in the current study. The other four metabolites of QDN were mainly distributed in the stomach and small intestine, followed by the liver or kidney, except salidroside was not found in the heart and brain. The maximum concentration of QDN in tissues at 1 h was 102 ± 22.1 μg/g specnuezhenide in the stomach. The average transfer rate of drug-to-tissue (Table 7) also corroborated the results of the metabolites in the tissue distribution (Figure 2).

**TABLE 7:** The average transfer rate of four metabolites from QDN to the tissues.

Tissues	Salidroside (%)	Calycosin-7-O-beta-glucoside (%)	Specnuezhenide (%)	Formononetin (%)
Heart	0	0.009 ± 0.002	0.012 ± 0.002	0.006 ± 0.001
Liver	0.368 ± 0.044	0.050 ± 0.012	0.080 ± 0.020	0.148 ± 0.046
Spleen	0.012 ± 0.003	0.008 ± 0.002	0.005 ± 0.001	0.011 ± 0.002
Lung	0.003 ± 0.000	0.008 ± 0.002	0.006 ± 0.001	0.010 ± 0.004
Kidney	0.061 ± 0.015	0.014 ± 0.003	0.012 ± 0.002	0.056 ± 0.014
Stomach	0.055 ± 0.012	0.171 ± 0.012	0.243 ± 0.053	0.342 ± 0.098
Intestine	0.028 ± 0.008	0.077 ± 0.020	0.194 ± 0.055	0.351 ± 0.090
Brain	0	0.016 ± 0.005	0.021 ± 0.003	0.008 ± 0.002

## 4 Discussion

As one a form of traditional therapy, Chinese medicines have attracted global attention in tumour treatment. To improve the quality and safety control of QDN, it is necessary to understand the transfer characteristics of representative markers in QDN to tissues. Therefore, content determination and tissue distribution studies are essential. In this study, five representative metabolites, i.e., salidroside (Gynostemmae pentaphylli Herba), calycosin-7-O-β-D-glucoside (Astragali Radix), specnuezhenide (Ligustri Lucidi Fructus), formononetin (Astragali Radix), and polyphyllin I (Paridis Rhizoma), were selected from QDN by referring to the assay of these botanical drugs in the Chinese Pharmacopoeia and the antitumour efficacy reported in the literature. Except for ruscogenin from Ophiopogonis Radix in QDN, which failed to show satisfactory ionization, the contents of the above five markers were successfully detected using the newly established LC‒MS method. Fortunately, the content results showed that the levels of the five metabolites were evenly distributed among the different batches of QDN samples. This result indicates that the manufacturing process of QDN is consistent, which ensures its efficacy and safety in clinical applications (Yang et al., 2023; Yu et al., 2023). From the perspective of material basis, this method provides a scientific basis for the QC of QDN.

On the other hand, traditional Chinese medicine compound preparations contain several botanical drugs and complex metabolites, and the antitumour mechanism of traditional Chinese medicine results from multitarget and multiorgan interactions (Li et al., 2015). Tumour cells cause tissue damage and affect multiple significant organs in the body, leading to multiple organ failures and worsening the patient’s condition. After oral absorption, drugs are distributed to various organs and tissues along with blood flow, which leads to an uneven distribution of drugs in tissues due to the differences in blood perfusion between different organs and different affinities between drugs and tissues.

Most pharmacokinetic and tissue distribution studies have suggested that oral administration at 0.5–1 h was the peak of the botanical drugs in rats (He et al., 2012; Yan et al., 2018; Wang et al., 2022), so the time point of 1 h administration was chosen for this tissue distribution study. This study found that the exposure of these metabolites was high in the stomach (administration site) and intestine (adsorption site), relating to the route of administration. Since the liver and kidney are the main metabolic and excretory organs with rich blood perfusion, the amount of markers from QDN was also higher than those in other tissues, which was in line with the pharmacokinetic rule of Chinese medicines (Shi et al., 2018). Except for formononetin, both calycosin-7-O-beta-glycoside and specnuezhenide are glucosides and hydrophilic. Nevertheless, their concentrations in the brain were as high as those in peripheral organs in our study, suggesting that they have the potential to cross the blood‒brain barrier. This is likely because calycosin-7-O-beta-glycoside can protect blood‒brain barrier integrity (Fu et al., 2014), and specnuezhenide is alkaline (p*K*a = 9.98 ± 0.15). Moreover, to our knowledge, this is the first time that the distribution of specnuezhenide in the brain is reported.

Usually, bioavailability is used to evaluate the efficiency of drug absorption in the blood (Chryssafidis et al., 2021). In this study, we analysed the average transfer rate of the drug to the tissues, which might be a new concept and an addition to the evaluation of drug bioavailability because it evaluates the absorption of the same drug into the tissues of the body instead of blood, at different formulations or routes of administration. Interestingly, it was found that the content distribution of the four metabolites in QDN was consistent with that of the metabolites in the tissues. For example, specnuezhenide had the highest concentration in QDN and also in tissues. However, after 1 h of administration, the highest transfer rate from QDN compound prescription to tissues was that of formononetin at 0.351% in the intestine, while that of specnuezhenide was only 0.194%.

There are limitations in this experiment. The efficacy of Chinese medicines is also related to the involvement of botanical metabolites in regulating intestinal flora metabolism (Li et al., 2021). For example, polyphyllin I in QDN was not detected in rat tissues, and whether it is related to absorption failure in the form of a prototype compound after intestinal flora action needs further experimental verification. In addition, it is a preliminary experiment that only detected the limited sample batches and the tissue distribution of QDN at a single time point, and the distribution rules at different time points in tissues and plasma are also worth further discussion.

## 5 Conclusion

This study established a novel HPLC-QQQ-MS/MS method for simultaneously determining five metabolites in QDN and their distribution in rat tissues. Batch-to-batch variation in QDN was stable for the five metabolites, but the distribution of the four metabolites in different tissues was different. The results of this study provide a basis for the QC of pharmacodynamic substances and the clinical application of QDN.

## Data Availability

The original contributions presented in the study are included in the article/Supplementary Material, further inquiries can be directed to the corresponding authors.
