# Supplementary material for: Establishment of a multicomponent quality control method and the transfer characteristics of five markers from Qidongning Formula to rat tissues by HPLC-QQQ-MS/MS
Source: Front Pharmacol. 2023 Dec 5;14:1310266. doi: 10.3389/fphar.2023.1310266 (PMC10728991; doi:10.3389/fphar.2023.1310266)
Supplement: Supplementary file 1 [file DataSheet1.docx]

**Supplementary materials**

**Establishment of a multicomponent quality control method and the transfer characteristics of five markers from Qidongning Formula to rat tissues by HPLC-QQQ-MS/MS**

Di Zhou ^1, #^, Jian-Ru Chen ^2, #^, Zi-Qi Yang ^4^, Ling Xu ^1, *^, Yu-Feng Huang ^3, *^

1. Department of Oncology, Yueyang Hospital of Integrated Traditional Chinese and Western Medicine, Shanghai University of Traditional Chinese Medicine, Shanghai, China
2. General Manager Office, Shanghai Tongjitang Pharmaceutical Co., LTD, Shanghai, China
3. State Key Laboratory of Traditional Chinese Medicine Syndrome, The Second Affiliated Hospital of Guangzhou University of Chinese Medicine, Guangzhou, Guangdong, China
4. College of Chinese Medicine, Guangzhou University of Chinese Medicine, Guangzhou, Guangdong, China

# Co-authors:

Di Zhou and Jian-Ru Chen contributed equally to this work.

* Corresponding authors:

Ling Xu, E-mail: xulq67@aliyun.com, and Yu-Feng Huang, E-mail: fongfonghuang@hotmail.com

**Method validation:**

*Specificity*

An aliquot of 100 μL of rat blank liver homogenate, a specific concentration of mixed reference solution including internal standard substance (IS) with liver homogenate, and liver homogenate after gavage administration were processed according to the method in 2.5.2, and representative MRM chromatograms of the analytes measured in different samples were obtained for methodological characterization. The metabolites were separated at baseline without interference from the matrix and IS.

*Linearity*

The method's linearity was evaluated by analysing the standards at five concentration levels in triplicate. The calibration curves were constructed by plotting the peak area (y) versus concentrations (x) of analytes using a multiple regression linear regression model. The correlation coefficient (r) should not be less than 0.999 for medicinal materials and 0.990 for biological samples. The lower limit of detection (LOD) was defined as the lowest amount of analyte that could be detected. The LOD was selected as the concentration with a signal-to-noise ratio ≥3:1. The lower limit of quantification (LOQ) was defined as the lowest concentration of the analyte in samples that could be determined with a signal-to-noise ratio ≥10:1.

For the tissue distribution study, blank liver tissue homogenate samples were accurately removed, and an appropriate amount of mixed reference solution and IS was added. The samples were treated according to the method in 2.5.2, and the supernatant was taken for mass spectrometry detection. Quality control (QC) and biological sample concentrations were calculated using the calibration curve regression equation.

*Precision* *and accuracy*

The precision was measured at one concentration level of standard solution six times continuously. Precision was defined as the measured peak area's relative standard deviation (RSD). The RSD determined at each concentration level should not exceed 5% for QDN compound prescription determination.

For the tissue distribution study, liver homogenate and QC solutions of high, medium, and low concentration were accurately absorbed and mixed, respectively, and processed according to the method in 2.5.2. Six samples were prepared in parallel and measured six times within one day for three consecutive days. Intraday and interday precision and accuracy were calculated. The default RSDs met the requirements within 20%.

*Repeatability*

The repeatability was determined by the QDN compound prescription testing sample, separated into six portions, and extracted separately, starting from the powdering to the end of HPLC-QQQ-MS/MS analysis. The RSD of each metabolite's content level should not be greater than 5% after determining concentrations in all six replicates.

*Stability*

The stability was determined by repeatedly testing the test solution of the QDN compound prescription stored at room temperature over 0, 2, 4, 6, 8, and 12 h in one day. The RSD of each metabolite's peak area should not be larger than 5%.

For the stability of the tissue distribution study, blank liver homogenate and QC solution of high, medium, and low concentration were accurately absorbed and mixed, respectively, and treated according to the method in 2.5.2. Three concentrations of QC samples were at room temperature for 6 h, at -20 °C for 30 d, and subjected to three freeze–thaw cycles (–80 °C  to ambient temperature) to investigate the stability. All the samples were prepared in parallel with 6 samples. The default RSDs meet the requirements of 20%.

*Recovery* *and Matrix Effects*

For the content determination study of QDN compound prescription, the recovery test was determined by spiking a certain amount of the sample with a known amount of the mixed standards repeatedly six times. The recovery was calculated by the equation below:

Recovery (%) = (total amount detected − original amount)/amount spiked × 100%

For the tissue distribution study, 100 μL of the blank liver homogenate was precisely absorbed and treated in three ways: high-, medium-, and low-concentration QC solutions were mixed with the blank tissue sample and treated according to the method in Section 2.5.2. The chromatographic peak area A was obtained. Blank tissue samples were directly treated according to the method in Section 2.5.2. Then, the tissue supernatant, after protein precipitation, was added to the mixed reference solution at the corresponding concentration. Then, the chromatographic peak area B was obtained by the same operation. The chromatographic peak area C was obtained by taking the above high-, medium-, and low-concentration QC solutions according to the method in Section 2.5.2. Extraction recovery = A/B×100%, matrix effect = B/C×100%.

The recovery rates of the five metabolites should be within the range of 95.0% to 105%, and the RSDs of the recovery rate of each metabolite should not be greater than 5% for compound prescription studies but should be in the range of 80.0% to 120% with RSDs ≤ 15% for tissue distribution studies.
